# High Air Humidity Causes Atmospheric Water Absorption *via* Assimilating Branches in the Deep-Rooted Tree *Haloxylon ammodendron* in an Arid Desert Region of Northwest China

**DOI:** 10.3389/fpls.2019.00573

**Published:** 2019-05-08

**Authors:** Xue-Wei Gong, Guang-Hui Lü, Xue-Min He, Binoy Sarkar, Xiao-Dong Yang

**Affiliations:** ^1^ Key Laboratory of Oasis Ecology, Xinjiang University, Urumqi, China; ^2^ College of Resources and Environmental Sciences, Xinjiang University, Urumqi, China; ^3^ Institute of Arid Ecology and Environment, Xinjiang University, Urumqi, China; ^4^ Department of Animal and Plant Sciences, The University of Sheffield, Sheffield, United Kingdom; ^5^ Future Industries Institute, University of South Australia, Mawson Lakes, SA, Australia

**Keywords:** foliar water uptake, rainfall pulse, deep-rooted woody plant, air relative humidity, ^18^O isotopic signatures

## Abstract

Atmospheric water is one of the main water resources for plants in arid ecosystems. However, whether deep-rooted, tomentum-less desert trees can absorb atmospheric water *via* aerial organs and transport the water into their bodies remains poorly understood. In the present study, a woody, deep-rooted, tomentum-less plant, *Haloxylon ammodendron* (C.A. Mey.) Bunge, was selected as the experimental object to investigate the preconditions for and consequences of foliar water uptake. Plant water status, gas exchange, and ^18^O isotopic signatures of the plant were investigated following a typical rainfall pulse and a high-humidity exposure experiment. The results showed that a high content of atmospheric water was the prerequisite for foliar water uptake by *H. ammodendron* in the arid desert region. After atmospheric water was absorbed *via* the assimilating branches, which perform the function of leaves due to leaf degeneration, the plant transported the water to the secondary branches and trunk stems, but not to the taproot xylem or the soil, based on the ^18^O isotopic signatures of the specimen. Foliar water uptake altered the plant water status and gas exchange-related traits, i.e., water potential, stomatal conductance, transpiration rate, and instantaneous water use efficiency. Our results suggest that atmospheric water might be a subsidiary water resource for sustaining the survival and growth of deep-rooted plants in arid desert regions. These findings contribute to the knowledge of plant water physiology and restoration of desert plants in the arid regions of the planet.

## Introduction

In arid ecosystems, water is the limiting factor on the ecological performance of plants (Kidron 2010; Yang et al., 2014a; Dai et al., 2015; Yang et al., 2017). In order to survive and grow in an extreme drought environment, desert plants develop special strategies to utilize all sorts of potential water resources (Yan et al., 2015). Atmospheric water is moisture that can be absorbed by the plant aerial organs, i.e., the unsaturated atmospheric water, clouds, fog, melting snow water, rainfall, and dewfall (Gotsch et al., 2014; Mayr et al., 2014; Eller et al., 2016; Pina et al., 2016; Wang et al., 2016a; Steppe et al., 2018). It is estimated that atmospheric water makes up 28–66% of the water input in the coastal prairie ecosystem of California (Corbin et al., 2005), and it can supply 74% water to plants for their growth and survival in arid desert regions (Kidron, 2000). However, owing to the differences in the absorptive pathways between atmospheric water and traditional water resources such as soil water and groundwater (Goldsmith, 2013), our understanding of how environmental conditions control plant aerial organs to absorb atmospheric water remains unclear.

The absorption of atmospheric water *via* plant aerial organs has been considered a crucial and universal phenomenon in previous studies (Schwerbrock and Leuschner, 2017). Although some recent studies have reported that the emergence of foliar water uptake might be related to the plant’s root functional type and water availability (Cassana et al., 2016; Ma et al., 2017), which environmental factors actually trigger this water absorption strategy is an ongoing debate among researchers (Goldsmith, 2013; Yan et al., 2015; Cassana et al., 2016; Wang et al., 2016b). In arid desert regions, shallow-rooted herbaceous plants usually absorb atmospheric water, whereas deep-rooted woody plants rely more on groundwater or deep soil water (Pan et al., 2016; Ma et al., 2017; Yang et al., 2017; Liu et al., 2018). The water absorption preferences of desert plants might be determined by the sustainability and magnitude of different water resources. It is generally believed that atmospheric water and groundwater are the two main types of water inputs in arid desert regions (Kidron, 2000; Golkarian et al., 2018). Atmospheric water is an unstable and scarce water source, while groundwater is a more stable and abundant water source (Dawson and Pate, 1996). Due to their perennial nature, deep-rooted woody plants in arid regions must increase carbon investments in their roots to obtain a large amount of groundwater to satisfy a higher water demand (Dai et al., 2015). In contrast, because they are annual or ephemeral plants, shallow-rooted herbaceous plants only absorb a small amount of atmospheric water to guarantee their growth (Zhuang and Ratcliffe, 2012; Yang et al., 2017). Thus, the difference in predilection for water sources between deep-rooted woody trees and shallow-rooted herbaceous plants may also depend on their water demand and the distinct accessibility of various water sources in arid desert regions (Zhuang and Ratcliffe, 2012; Cassana et al., 2016; Yang et al., 2017). Desert deep-rooted woody trees generally have a considerably higher biomass than shallow-rooted herbaceous plants and therefore tend to utilize all categories of potential water resource inputs to sustain their growth and survival in water-limited environments. Additionally, simulation experiments have suggested that the emergence of uptake of atmospheric water by desert plants, to some extent, depends on the magnitude of atmospheric water (Zhuang and Ratchiffe, 2012; Yang et al., 2017). In other words, high humidity might be the precondition for the absorption of atmospheric water *via* aerial organs for the deep-rooted plants in arid ecosystems. However, whether deep-rooted desert trees can absorb atmospheric water and the assumption that the atmospheric water content is closely related to atmospheric water uptake *via* the aerial organs of deep-rooted desert plants have not yet been properly assessed in previous studies (Yan et al., 2015; Wang et al., 2016b).

The soil-plant-atmosphere continuum (SPAC) and traditional water transport theories assume that water moves from the soil through a plant and then out into atmosphere due to the difference in water potential (*Ψ*) (Philip, 1966). Within the SPAC, the water potential gradually decreases from soil to atmosphere. However, if atmospheric water is absorbed by plant aerial organs, water migrates from the atmosphere to trunk xylem, and even to root xylem, and finally to rhizosphere soils (Eller et al., 2013; Cassana et al., 2016). This indicates that there would be a reversal of water potential gradient from the SPAC and traditional water transport theories. Hence, there would exist two processes in plants: (1) water moves from a higher *Ψ*_atmosphere_ to lower *Ψ*_stem_ through the foliar absorption of atmospheric water, while water also simultaneously rises from a higher *Ψ*_soil_ to lower *Ψ*_stem_, thus plants obtain water from two directions; and (2) water moves from a higher *Ψ*_atmosphere_ to a lower *Ψ*_soil_ only through the foliar absorption of atmospheric water (Goldsmith, 2013). In recent studies, the inverse water potential gradient in plants has been reported under artificial controlled conditions (Yan et al., 2015), but no research has found this phenomenon under field conditions. Additionally, there is no explicit conclusion on which factors control foliar water uptake in plants.

*Haloxylon ammodendron* (C.A. Mey.) Bunge (Chenopodiaceae) is a xerophytic woody dominant species in the arid deserts of Asia (Xu et al., 2016), which is a deep-rooted plant with many xeromorphic characteristics in its leaves and roots to adapt to drought (Huang et al., 2003; Dai et al., 2015). It has been reported that the assimilating branches of *H*. *ammodendron* are sensitive to changes in water inputs *via* physiological performance (Xu et al., 2007; Yang et al., 2014a). In this species, transpiration and carbon assimilation occur at the branch level due to leaf degeneration. Additionally, atmospheric water constitutes a large proportion of the available water resources in the arid desert region (Kidron, 2000). Thus, it is possible that the assimilating branches of *H*. *ammodendron* might absorb atmospheric water.

In this study, some plant water status and gas exchange traits of *H. ammodendron* were assessed following a natural rainfall pulse and in an artificial high-humidity exposure experiment to monitor the processes of absorption and transport of atmospheric water from the assimilating branches to the stems of *H. ammodendron*. Here, we hypothesized that (1) the high content of atmospheric water is the prerequisite for foliar water uptake by the deep-rooted plant in arid desert regions, and (2) the absorbed water will cause changes in water status and gas exchange and will cause water potentials to reverse direction from the SPAC and traditional water transport theories.

## Materials and Methods

### Study Site and Plant Species

The study site is located in the Ebinur Lake Wetland National Nature Reserve (82°36′–83°50′E, 44°30′–45°09′N) in the southwestern part of Gurbantunggut Desert, Xinjiang Uygur Autonomous Region, China. Consistent with a typical continental climate, this region is extremely dry and has scarce rainfall and frequent dust storms (He et al., 2015). Additionally, winters and summers are long, whereas springs and autumns are short in this region. The annual sunshine hours reach approximately 2,800 h. The annual precipitation is less than 100 mm, and rainfall events of ≤ 5 mm and > 10 mm account for 87.5 and 4.3% of the total rainfall, respectively (Zheng et al., 2012). The annual potential evaporation is more than 1,600 mm (Yang et al., 2017). The area is dominated by sunny days throughout the year; hence, precipitation is just an occasional event. The groundwater level is 1.50–2.30 m, and groundwater is the main water source for local plants and the desert ecosystem (Yang et al., 2014b; Ma et al., 2017).

*H. ammodendron* is a dominant woody species in the arid desert region, which grows naturally in a variety of habitats in the Asian and African deserts, i.e., gravel desert, clay desert, saline land, fixed and semi-fixed sandy lands (Tobe et al., 2000). Owning to its ecological roles in combating desertification and maintaining the desert forest community, *H. ammodendron* is considered a sustainer of arid desert ecosystems (Zhuang and Zhao, 2017). In the present research, 4 km^2^ typical plots of *H. ammodendron* forest were first chosen as our experimental plots. Then, some young *H. ammodendron* individuals were randomly selected as our experimental subjects. Here, the selection of young *H. ammodendron* was needed due to the maneuverability of the high-humidity exposure experiment involving the plant species (see the “Experimental design” section below).

### Experimental Design

To test our first hypothesis that the high content of atmospheric water is the prerequisite for foliar water uptake, a natural rainfall pulse and a high-humidity exposure experiment were conducted in this study. Precipitation in this arid desert is very scarce and is only worth 10% of evapotranspiration; hence, the atmospheric water content is low under traditional sunny conditions (e.g., the daily average air relative humidity on sunny days ranges from 25 to 50% in this region). However, a high content of atmospheric water can be reached during and following a rainfall event, which might result in direct water uptake *via* aerial organs. Similarly, a high-humidity exposure experiment could raise the atmospheric water content to a high level, which could also induce the emergence of foliar water uptake by the plant species. It is believed that the content of atmospheric water can be represented as the air relative humidity (RH); thus, our experimental objectives can be described as defining the causality between RH and foliar water uptake.

**Figure 1:**
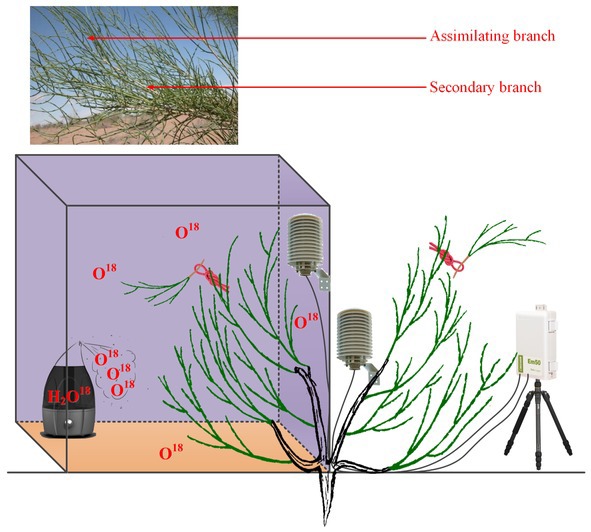
Design of the high-humidity exposure experiment.

For the natural rainfall pulse experiment, three traditional sunny days (12th, 13th, and 16th July 2016) and a natural rainfall event (20th July 2016) were randomly selected as a humidity experiment to test the differences in water potentials between plant organs as well as the differences in RH between rainy and sunny days. The traditional sunny day was characterized by less than 10% cloud cover and the peaks of unimodal photosynthetic photon flux density curves greater than 1,600 mmol m^−2^ h^−1^. The selected rainfall event occurred at 16:11–16:30 (local time, the same below) and lasted for 20 min. The total rainfall amount reached 3.56 mm. It was reported that the typical rainfall in the Gurbantunggut Desert is approximately 3–5 mm; thus, our selected rainfall was a typical rainfall pulse in this region (Zheng et al., 2012). Within the rainy and sunny days, atmospheric water conditions (RH) and soil volumetric water content (Svwc) (30 cm) were monitored using a VP-4 sensor (Decagon Devices Inc., USA) and a 5-TE probe (Decagon Devices Inc., USA), respectively. The interval of data collection on both instruments was 1 min, and the data were recorded with a data logger (EM50, Decagon Devices Inc., USA). Additionally, water potentials in the assimilating branches (*Ψ*_ab_) and secondary branches (*Ψ*_sb_) of *H. ammodendron* on rainy and sunny days were measured in this experiment. The occurrence of an inverse water potential gradient (*Ψ*_ab_ > *Ψ*_sb_) on the rainy day was regarded as confirmation of our first hypothesis (Goldsmith, 2013). The assimilating branches refer to the tender and succulent current-year branches (phylloclades) that take over the function of gas exchange from the degenerated leaves, thus minimizing evaporative water loss (Huang et al., 2003). In the present study, the secondary branch is defined as a twig on which assimilating branches converge (the assimilating branch was regarded as the first level branch). It is easy to distinguish the assimilating branch and the secondary branch on the basis of their morphology because their colors are glossy green and greyish white, respectively (Figure 1).

For the high-humidity exposure experiment, a young *H. ammodendron* individual plant with a basal diameter of 4.2 cm roughly divided into two main branches was selected as our experimental subject (Figure 1). During the experiment, the aboveground part of the plant was divided into two types of branches: one type of branch was the treatment that was enclosed in a humidity chamber, while the other type was exposed to natural conditions and considered the control (Figure 1). The humidity chamber was built of polymethylmethacrylate (PMMA) and had 1.2 m × 1.2 m × 1.5 m dimensions. The joints between PMMA sheets were sealed with transparent adhesive tape. Additionally, in order to eliminate fluxes of water and heat between soil and air in the chamber, a multilayer low density polyethylene film (LDPF) was placed on the bottom of the humidity chamber (Figure 1). Atmospheric humidity inside the chamber was controlled by an ultrasonic humidifier placed on the LDPE, and RH was maintained above 90% by turning the humidifier on or off (Figure 1). In addition, in this experiment, a pocket weather meter (Model 5000, KestrelMeters, USA) was hung in the chamber and used to quickly obtain RH status in the chamber. At the same time, two VP-4 sensors (Decagon Devices Inc., USA) were also hung on the treatment and control branches to record the real-time changes in RH and air temperature (T) inside and outside the chamber. RH and T were measured every 1 min and recorded with a data logger (EM50, Decagon Devices Inc., USA) (Figure 1). In this experiment, the high-humidity exposure experiment was conducted for 8 h from 20:00 on 25th July 2016 to 04:00 on 26th July 2016. Within the experimental period, *Ψ*_ab_ and *Ψ*_sb_ of *H. ammodendron* outside and inside the chamber were also measured. In addition, since the humidifying water was ^18^O labeled (*c*. 3008.52‰ *δ*^18^O) in the high-humidity exposure experiment (Figure 1), the emergence of the labeled ^18^O in assimilating branches and other organs can also demonstrate our first hypothesis. Here, the ^18^O-labeled water was composed of a mixture of groundwater (*c*. −14.39‰ *δ*^18^O) and water enriched in ^18^O (98%, Shanghai Research Institute of Chemical Industry, CHN). Because of the constraints of budget and experimental operability, it was difficult to implement multiple high-humidity chambers in the field to test the atmospheric water absorption and transport within the plant body. Therefore, the high-humidity exposure experiment was conducted only once in this study (Yan et al., 2015; Wang et al., 2016b).

To test the second hypothesis that the absorbed water will cause a variability in the water transport process and cause an inversion in water potentials from the SPAC and traditional water transport theories, the difference between *Ψ*_ab_ and *Ψ*_sb_ of *H. ammodendron* was measured in both the rainfall pulse and high-humidity chamber experiments. In addition, the differences in the values of *δ*^18^O among the different plant organs and soil were measured in the high-humidity exposure experiment.

In this study, the outstanding characteristics of foliar water uptake would probably be impeded by root water uptake. Thus, some inferior shoots (containing assimilating branches and secondary branches) of the treatment and control branches were cut. All the end cuts were immediately sealed with petrolatum, and the excised inferior shoots were then hung inside and outside the chamber to distinguish the effects of root and foliar water uptake (Figure 1). Therefore, the entire high-humidity exposure experiment was composed of four treatments: inside-attached, inside-detached, outside-attached, and outside-detached. “Attached” represents the samples connected to the main stem, and “detached” represents the samples cut from the main stem; “inside” and “outside” represent the inside and outside of the high-humidity chamber. In addition, since the foliar water uptake might cause some changes in gas exchange traits, such as stomatal conductance (*g*_s_, mol CO_2_ m^−2^ s^−1^), transpiration rate (*E*, mmol H_2_O m^−2^ s^−1^), and instantaneous water use efficiency (WUEi, μmol CO_2_ mmol H_2_O), differences in these traits among the four treatments were also tested in this study.

### Measurements

In the rainfall pulse experiment of this study, five young individual *H. ammodendron* plants with similar heights and basal diameters (~1.5 m height and ~5 cm basal diameter) were randomly selected as subjects to test the differences in water potential between assimilating branches and secondary branches. Immediately after a natural rainfall pulse (20th July 2016) and on three representative sunny days (12th, 13th, and 16th July 2016), one shoot (containing the assimilating and the secondary branches) of each individual plant was collected (all the end cuts caused by sampling were immediately sealed with petrolatum) and sealed immediately in a plastic bag containing moist paper towels. The samples were subsequently kept in a cooler until the water potential was determined. The measurements of *Ψ*_ab_ and *Ψ*_sb_ were taken using a dew point water potential instrument (WP4C, Decagon Devices, Pullman, WA, USA). The interval of water potential measurements was 2 h, and the measurement was conducted from 20:00 to 4:00.

During the process of humidifying in the high-humidity exposure experiment, *Ψ*_ab_ and *Ψ*_sb_ were also measured at 2 h intervals from 20:00 to 04:00. For the samples in the chamber, we opened the door of the chamber every 2 h to cut the assimilating and the secondary branches to determine their differences in water potential. To decrease the effects of opening on the maintenance of high humidity in the chamber, the duration of each opening was less than 15 min. All samples from the inside and outside of the chamber with three replications were measured at each time (all the end cuts caused by sampling were immediately sealed with petrolatum). To test the effect of foliar water uptake on gas exchange traits, *g*_s_, *E*, and WUEi of three assimilating branches from inside and outside of the chamber were measured at the end of the high-humidity exposure experiment (4:00) using a portable photosynthesis system (LI-6400XT, Li-COR, Inc., Lincoln, NE, USA). After that, three samples of the assimilating branches, secondary branches, and trunk xylem from inside and outside of the chamber [the samples collected from the inside of the chamber were washed with tap water and dried with paper towels to avoid potential isotopic contamination of labeled water condensed on the surface of samples (Eller et al., 2013)], as well as three samples of root xylem, rhizosphere soil, and bulk soil were carefully collected to identify the transport direction of ^18^O water through the atmosphere, the assimilating branches, stems, roots, rhizosphere soils, and bulk soils. Meanwhile, three samples of taproot xylem, rhizosphere soil, and bulk soil of adjacent natural *H. ammodendron* plants were collected in order to test whether the ^18^O-labeled water was transported from the atmosphere to the soil *via* plant stem. Here, the significant difference in *δ*^18^O signature between humidifying and natural samples was used to illustrate the above water transport mechanisms. Following collection, all isotope samples were rapidly put in vials and sealed with parafilm. Subsequently, all samples were placed in a −20°C frozen box. The *δ*^18^O measurement was conducted at the Fukang Desert Ecosystem Observation and Experiment Station, Chinese Academy of Sciences. Specifically, water was extracted from the plant tissues and soil samples using a vacuum extraction instrument (LI-2000, Lica United, Beijing, CHN), and the measurement of oxygen isotopic compositions was conducted using an isotope ratio infrared spectroscopy (IRIS) analyzer-the Liquid Water Isotope Analyzer (LWIA, DLT-100, Los Gatos Research Inc., Mountain View, CA, USA). The analytical precision of individual measurements was ± 0.25‰ for *δ*^18^O (Dai et al., 2015). The isotopic abundance was expressed in delta notation (*δ*) in parts per thousand (‰) as *δ* = (*R_sample_*/*R_standard_* − 1) × 1,000, where *R_sample_* and *R*_standard_ are the molar ratios of heavy to light isotope in the sample and the international standard (Vienna Standard Ocean Water for ^18^O/^16^O), respectively.

### Data Analysis

In this study, the paired Student’s *t*-test was used to test the differences in RH, T, Svwc, and *Ψ*_ab_ among different atmospheric humidity levels. In addition, the unpaired Student’s *t*-test was used to indicate the differences in nocturnal *Ψ*_ab_ and *Ψ*_sb_ at each measurement time on rainy and sunny days. All statistical analyses were conducted using SPSS 17.0 (SPSS Inc., Chicago, USA). All data were tested for normality and variance constant, and *p* < 0.05 was considered to be statistically significant.

## Results

### Atmospheric Water Uptake *via* Assimilating Branches Following a Rainfall Pulse

**Figure 2:**
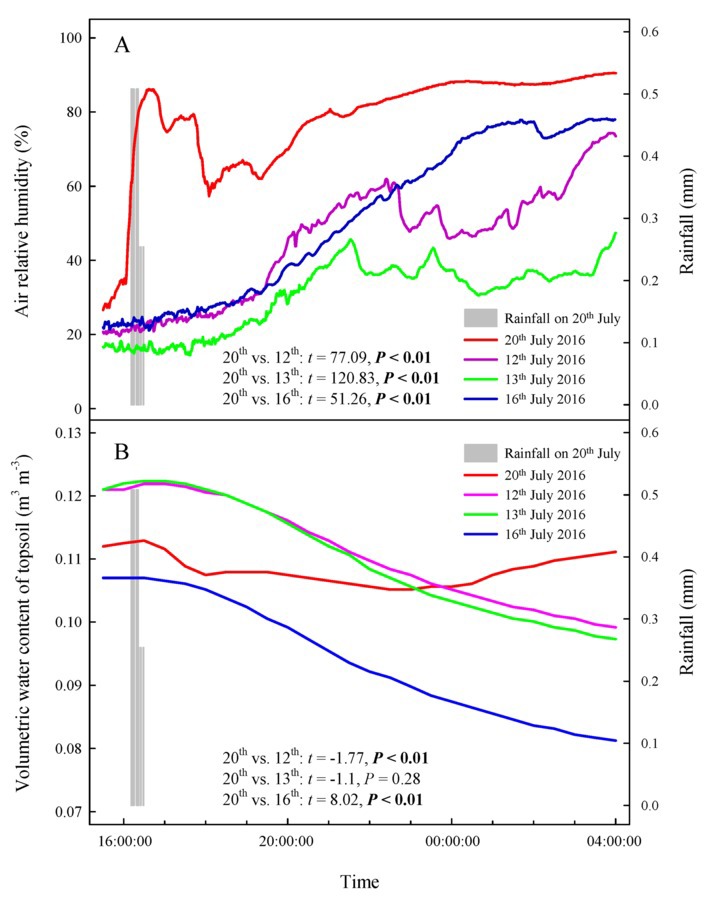
Changes in atmospheric and soil moisture on a rainy day and three sunny days. **(A)** air relative humidity, **(B)** volumetric water content of topsoil. The bars represent a rainfall pulse at 16: 11–16: 30 on 20th July 2016. The red line represents the changes in atmospheric and soil moisture following a rainfall pulse on 20th July 2016. The purple, green and blue lines represent the changes in atmospheric and soil moisture on three representative sunny days (12th, 13th, and 16th July 2016), respectively. The *t*’s and *p*’s are the results of the paired Student’s *t*-test for the variability of atmospheric and soil moisture between rainy and sunny days.

**Figure 3:**
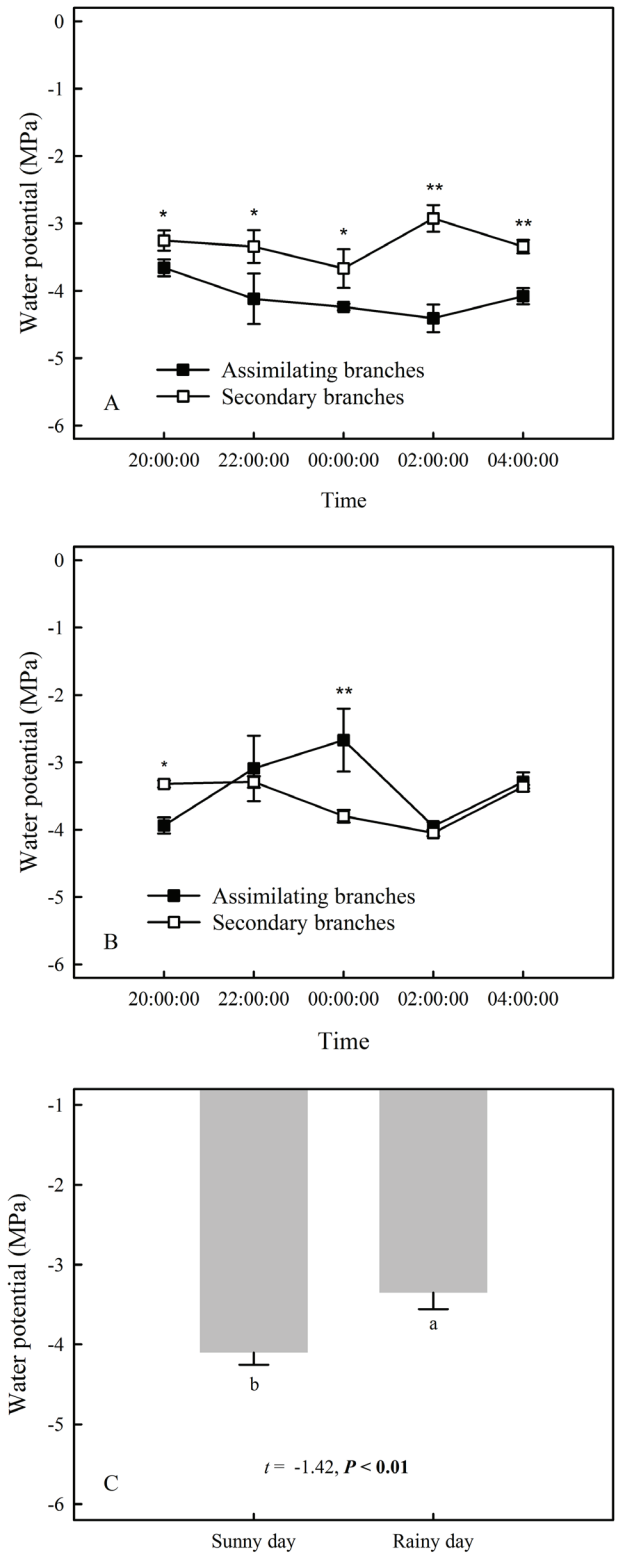
The compared relationship of the assimilating branches to the secondary branches of *H. ammodendron* in water potential on the sunny **(A)** and rainy days **(B)**, as well as the nocturnal mean water potential of assimilating branches between sunny and rainy days **(C)**. The asterisks at each measurement time in panels **(A)** and **(B)** represent the significant differences in water potential between the assimilating branches and the secondary branches (unpaired Student’s *t*-test; ^*^, *p* < 0.05; ^**^, *p* < 0.01). Different lowercase letters on the top of the bars in panel **(C)** represent the significant difference in nocturnal mean water potential of assimilating branches between rainy and sunny days, the *t*’s and *p*’s are the results of the paired Student’s *t*-test. Values are shown as the mean ± SE.

Our results showed that the RH values at any time during the rainfall pulse were significantly higher than those during the three typical sunny days (Figure 2A) (*p* < 0.01). The rainfall pulse also had a long-term effect, maintaining RH at a high level for 12 h after the rainfall (Figure 2A). In summary, the rainfall pulse increased the amount and availability of atmospheric water and had a great influence on the Svwc of the topsoil (Figure 2B). During the three typical sunny days, *Ψ*_sb_ > *Ψ*_ab_ was maintained at each measurement occasion at night (Figure 3A). However, the inverse water potential gradient between *Ψ*_sb_ and *Ψ*_ab_ occurred during the rainy day (*Ψ*_ab_ > *Ψ*_sb_) (Figure 3B). Specifically, after the rainfall pulse, *Ψ*_sb_ was lower than *Ψ*_ab_ from 22:00 to 04:00, whereas the other periods showed the opposite trend (Figure 3B). Additionally, the nocturnal mean value of *Ψ*_ab_ was higher on the rainy day than on the sunny days (Figure 3C).

### Atmospheric Water Uptake *via* Assimilating Branches in the High-Humidity Exposure Experiment

**Figure 4:**
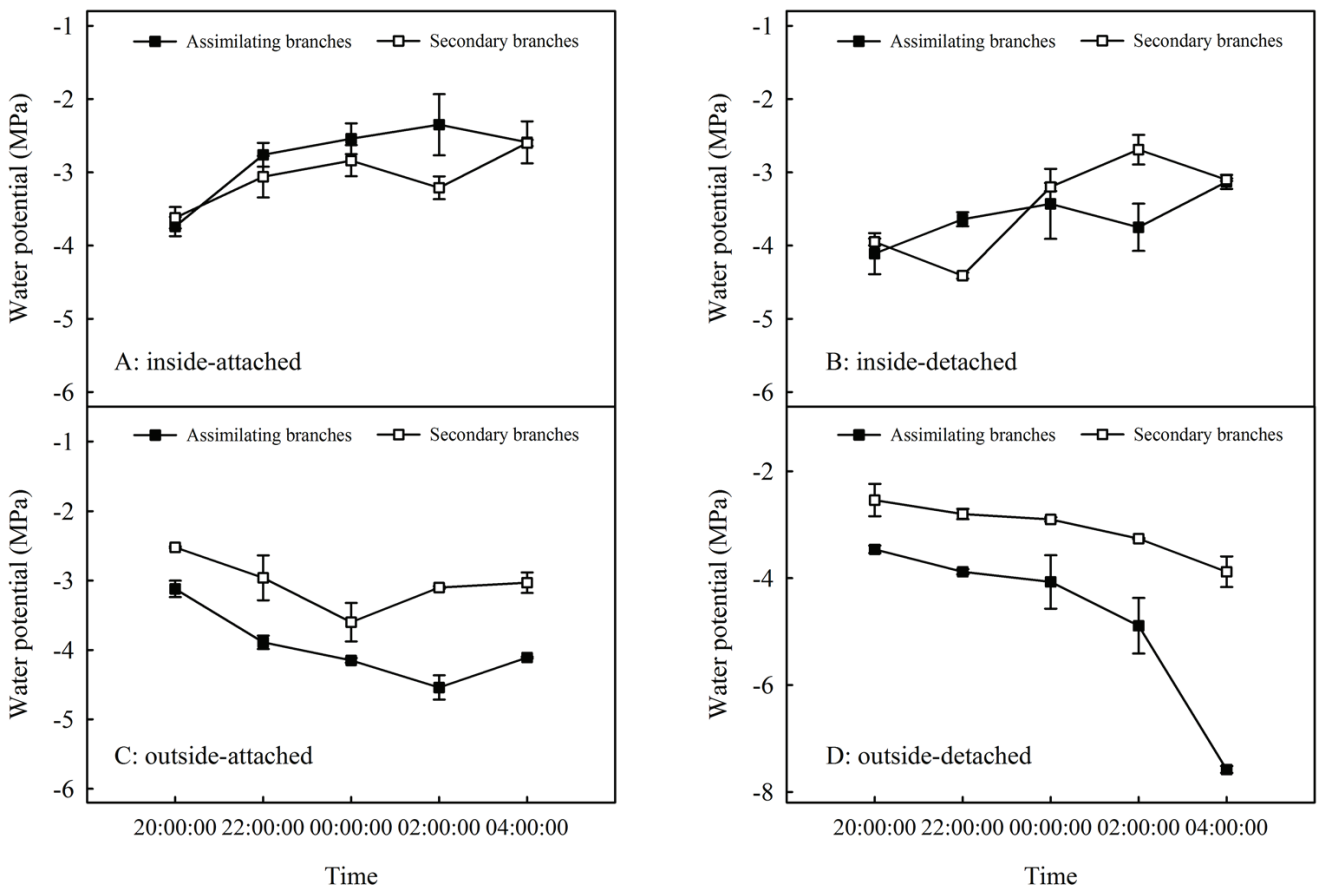
Relationships in water potential between the assimilating branches and the secondary branches of *H. ammodendron* in inside-attached **(A)**, inside-detached **(B)**, outside-attached **(C)** and outside-detached **(D)** treatment in the high-humidity exposure experiment. “Attached” represents the samples connected to the main stem, and “detached” represents the samples cut from the main stem; “inside” and “outside” represent the inside and outside of the high-humidity chamber. Values are shown as the mean ± SE.

**Figure 5:**
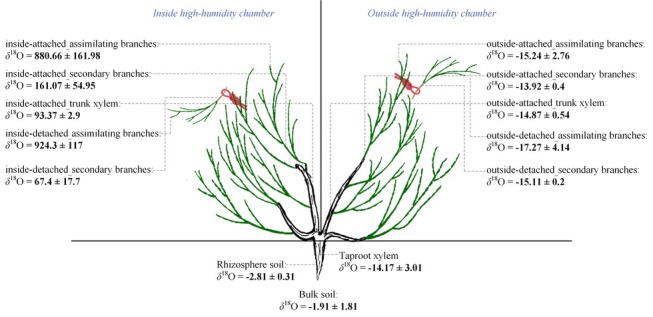
Differences in the value of *δ*^18^O across the assimilating branches, secondary branches, trunk stem, taproot xylem and soil inside and outside of the chamber, as well as in the same plant organs between the inside and outside of the chamber of the high-humidity exposure experiment. “Attached” represents the samples connected to the main stem, and “detached” represents the samples cut from the main stem; “inside” and “outside” represent the inside and outside of the high-humidity chamber. Values are shown as the mean ± SE.

Relationships between *Ψ*_ab_ and *Ψ*_sb_ differed among the four experimental treatments in the high-humidity exposure experiment (Figure 4). Specifically, *Ψ*_ab_ > *Ψ*_sb_ occurred in inside-attached and inside-detached treatments (Figures 4A,B), whereas outside-attached and outside-detached treatments showed the opposite result (*Ψ*_sb_ > *Ψ*_ab_) (Figures 4C,D). The duration of *Ψ*_ab_ > *Ψ*_sb_ in the inside-attached treatment was longer than that in the inside-detached treatment (Figures 4A,B). All observed times showed *Ψ*_sb_ > *Ψ*_ab_ in both the outside-attached and outside-detached treatments (Figures 4C,D). In addition, the value of *δ*^18^O in the assimilating branches of the inside-attached treatment was higher than that in the outside-attached treatment; meanwhile, the assimilating branch *δ*^18^O of the inside-detached treatment was higher than that of the outside-detached treatment (Figure 5).

### Difference in Water Transport Between the Inside and Outside of the Chamber

**Figure 6:**
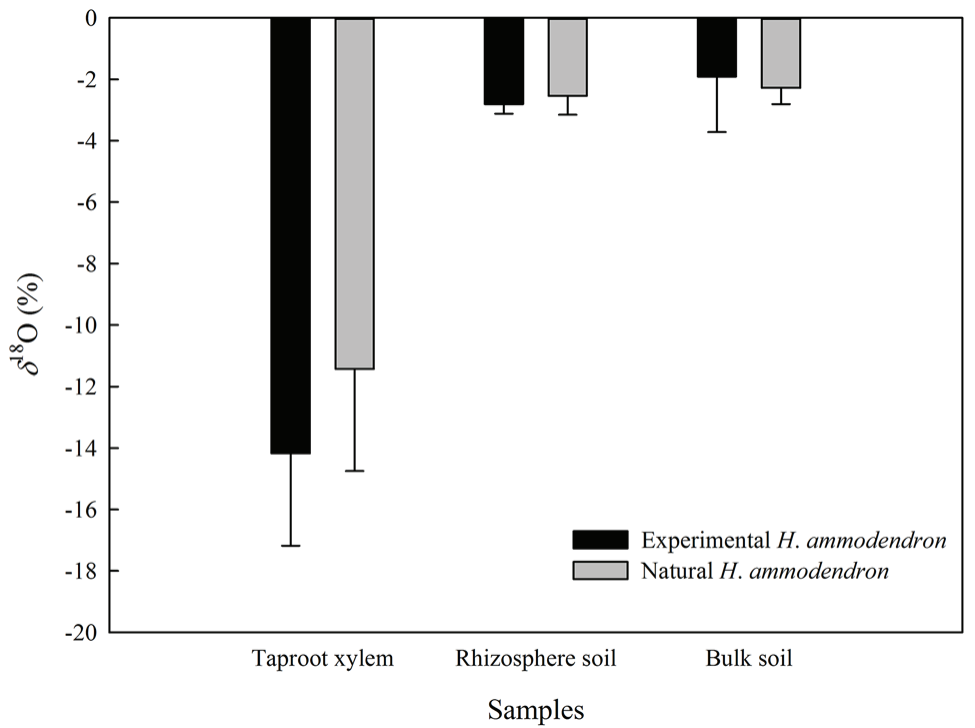
Differences in the value of *δ*^18^O of the taproot xylem, rhizosphere soil and bulk soil between the experimental and the natural *H. ammodendron* individuals. Values are shown as the mean ± SE.

In the inside-attached treatment, the value of *δ*^18^O was the highest in assimilating branches, intermediate in secondary branches, and the lowest in trunk xylem (Figure 5). In the inside-detached treatment, although the water pathway was cut-off from assimilating branches to trunk xylem, our results showed that the value of *δ*^18^O was substantially higher in the assimilating branches than in the secondary branches (Figure 5). In contrast, the values of *δ*^18^O were not different among the assimilating branches, secondary branches or trunk xylem in the outside-attached and outside-detached treatments (Figure 5). In addition, the values of *δ*^18^O in assimilating branches, secondary branches, and trunk xylem from outside the humidity exposure chamber were lower than those from inside the chamber (Figure 5). The values of *δ*^18^O in taproot xylem, rhizosphere soil, and bulk soil were not different between the humidifying and natural samples (Figure 6).

### Response of Plant Water Status and Gas Exchange to Atmospheric Water Uptake

**Figure 7:**
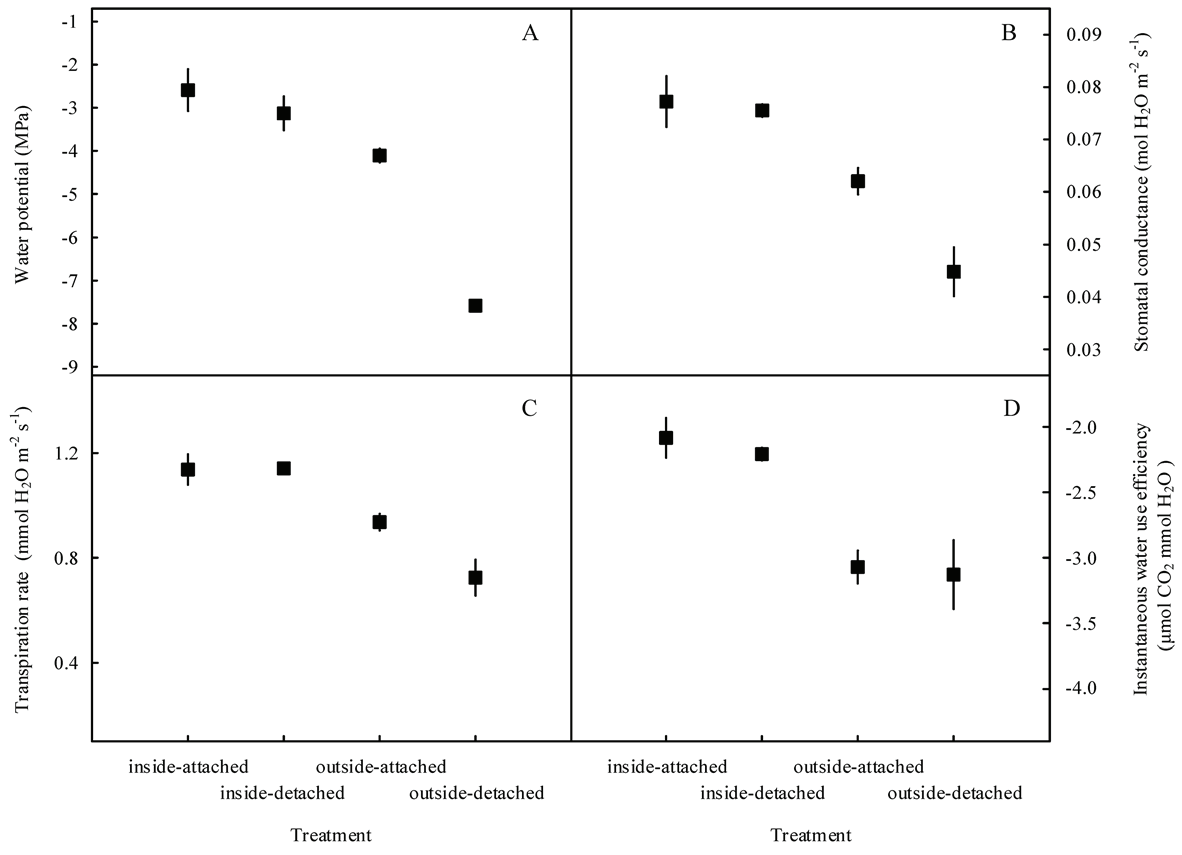
Variations in water status and gas exchange-related traits of the assimilating branches among the four experimental treatments in the high-humidity exposure experiment. “Attached” represents the samples connected to the main stem, and “detached” represents the samples cut from the main stem; “inside” and “outside” represent the inside and outside of the high-humidity chamber. Water status and gas exchange-related traits include water potential **(A)**, stomatal conductance **(B)**, transpiration rate **(C)**, and instantaneous water use efficiency **(D)**. Values are shown as the mean ± SE.

**Figure 8:**
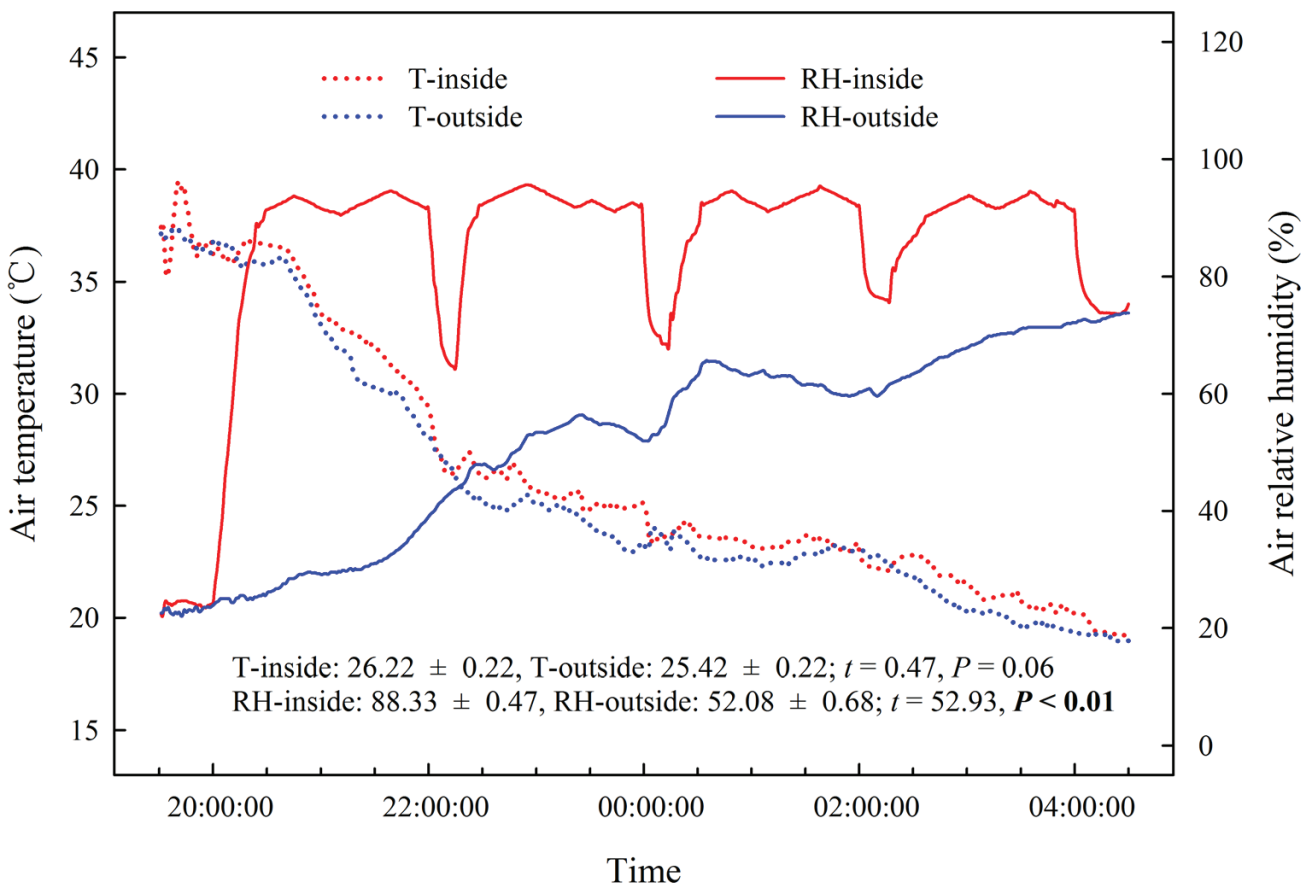
Changes and differences in air temperature (T) and air relative humidity (RH) during the experimental time and between the inside and outside of the chamber of the high-humidity exposure experiment. Experimental time ranges from 20:00 on 25th July to 4:00 on 26th July 2016, and samples were collected at 20:00, 22:00, 0:00, 2:00 and 4:00. The *t*’s and *p*’s are the results of the paired Student’s *t*-test. Values are shown as the mean ± SE.

Our results showed that the predawn *Ψ*, *g*_s_, *E*, and WUEi in the assimilating branches were higher inside the high-humidity exposure chamber than they were outside the chamber (Figure 7). Physiological measurements indicated that both foliar water uptake and root water uptake had great influence on predawn *Ψ*, *g*_s_, and *E* values (Figures 7A–C). Interestingly, the variability of WUEi was seemingly only influenced by foliar water uptake because the attached and detached treatments had similar WUEi values both inside and outside the chamber (Figure 7D). In addition, RH was significantly higher inside the high-humidity exposure chamber than outside (*p* < 0.05), whereas T did not differ between the inside and outside of the chamber (Figure 8). This indicated that the difference in plant water status and gas exchange inside and outside the chamber was not affected by the T but was the result of the difference in RH.

## Discussion

### Atmospheric Water Uptake *via* Assimilating Branches

In arid desert regions, rainfall is considered the most readily obtainable atmospheric water for plants. A large proportion of rainfall is intercepted by plant foliage and is generally assumed to evaporate back into the atmosphere or fall onto the topsoil in arid desert ecosystems (Breshears et al., 2008). However, our results showed that a reverse relationship in water potential (*Ψ_ab_* > *Ψ_sb_*) appeared after a rainfall pulse (Figure 3B), which indicated that the assimilating branches directly absorbed rainfall water from the atmosphere (Goldsmith, 2013; Yan et al., 2015). It is believed that the availability of atmospheric water might be the decisive condition of foliar water uptake in arid desert regions (Zhuang and Ratcliffe, 2012; Yang et al., 2017). In this regard, RH might have a significant relationship with the occurrence of foliar water uptake. The results of the present study showed that the RH increased significantly after a rainfall event (Figure 2A) and then caused *Ψ_ab_* > *Ψ_sb_* (Figure 3B). This result suggested that the high RH level was the prerequisite for foliar water uptake in this arid desert region. Similar results were also found in previous studies, e.g., the pseudostem of *Vellozia flavicans* and leaves of *Juniperus* absorbed atmospheric water after rainfall events (Oliveira et al., 2005; Breshears et al., 2008). In addition, the relationship between RH and the occurrence of foliar water uptake was also confirmed in our high-humidity exposure experiment. Our results demonstrated an inverted water potential gradient between the assimilating branches and secondary branches (*Ψ_ab_* > *Ψ_sb_*) (Figures 4A,B). We also found that the labeled ^18^O water appeared in varying amounts in the assimilating and secondary branches inside the humidity chamber, whereas the ^18^O abundances did not differ between the two types of branches outside the chamber (Figure 5). These results also indicated that the assimilating branches absorbed atmospheric water at high RH levels.

A rainfall event of approximately 6–12 mm was considered effective precipitation for *H. ammodendron* in the arid desert region because this amount of rainfall could overcome crown interception to supply soil water and could trigger a cascade of plant physiological responses (Yang et al., 2014a). However, our study showed that the assimilating branches of *H. ammodendron* absorbed atmospheric water through assimilating branches at 3.56 mm rainfall (Figure 3B). This result suggested that even inappreciable precipitation, such as light rain, dew, and fog, might not directly replenish soil moisture but could play an important role in plant water physiology by rehydrating plant tissues (Dawson, 1998; Burgess and Dawson, 2004). Additionally, our study found that foliar water uptake appeared at high RH levels, which was not observed during sunny days. However, exactly what level of RH would control the switch to atmospheric water uptake still remains unclear and warrants continuous research in the future.

Previous studies indicated that the tomentum on plant aerial organ surfaces was the location of atmospheric water absorption by the plant (Vitarelli et al., 2016). It was reported that plant aerial organs without the tomentum would not absorb water from the atmosphere (Grammatikopoulos and Manetas, 1994; Zhuang and Ratcliffe, 2012). However, our results showed an opposite trend: the tomentum-less assimilating branches of *H. ammodendron* were also able to absorb water from the atmosphere under certain conditions (Figures 4,5). These results suggest that there might be other specialized structures on the surfaces of assimilating branches of *H. ammodendron* that enable the plant to absorb water from the atmosphere. Previous scanning electron microscopy studies indicated that the epidermal structure of the assimilating branches of *H. ammodendron* was uneven and crinkled (Liu et al., 2016). This is conducive to the retention of atmospheric water on the surface of plants and thus might be advantageous in absorbing water from the atmosphere (Eller et al., 2013; Pan et al., 2016). In addition, a recent fluorescence labeling study indicated that the aqueous pores throughout cuticles could allow water transport from the atmosphere into the mesophyll of assimilating branches of *H. ammodendron* (Wang et al., 2016b). These aqueous pores were formed from the continuous permanent dipoles and ionizable groups across cuticles of the leaf surface under the conditions of high humidity and water deposition (Schönherr, 2006). It has been shown that direct water uptake by distal leaves from moist air might be mediated by the mucilage layers on the leaf surface and/or the epistomatal mucilage plugs set into the substomatal cavity due to the water binding capacity of acid mucopolysaccharides (Zimmermann et al., 2004, 2007; Westhoff et al., 2009). In many species, mucilage cells were also detected in the inner walls of xylem conduits, and the xylem mucilage was considered to play important roles in shifting water from the mucilage layers and epistomatal plugs to the xylem vessels *via* the apoplast-symplast pathway (Zimmermann et al., 2007; Mastroberti and de Araujo Mariath, 2008). Deng et al. (1998) proved that the assimilating branches of *H. ammodendron* contain abundant mucilage cells; thus, mucilage cells might be a potential location for atmospheric water uptake by assimilating branches and an approach transporting atmospheric water from the assimilating branches to the stem. Therefore, external microstructures, such as rough epidermis, aqueous pores, and mucilaginous substances, might be potential pathways for atmospheric water absorption in tomentum-less plants such as *H. ammodendron* (Zimmermann et al., 2007; Liu et al., 2016; Pan et al., 2016; Wang et al., 2016b).

### Effect of Foliar Water Uptake on Water Transport and Physiological Performance

Root water uptake is considered the primary method by which plants absorb water in traditional plant physiology literature (Emery, 2016). Water transport from soil into the atmosphere in the SPAC framework dominated the scientific discussion on plant water movement for many years (Philip, 1966). However, whether absorbed atmospheric water can transport from leaves to the soil remains unclear (Eller et al., 2013; Goldsmith, 2013; Yan et al., 2015; Emery, 2016). The current study showed that the values of *δ*^18^O differed inside and outside of the high-humidity chamber (Figure 5), as well as among the assimilating branches, secondary branches and stems inside the high-humidity chamber (Figure 5). Additionally, the values of *δ*^18^O in the taproot xylem, rhizosphere soil, and bulk soil did not significantly differ between the humidifying and natural samples (Figure 6). These results suggested that the absorbed atmospheric water was transported from the assimilating branches to the trunk stem, but not to the taproot xylem. The above might be determined by the interrelationship between foliar water uptake, root water uptake, and water storage in trunk stems. The opposite direction of water transport during foliar water absorption and transpiration might hinder the downward movement of water uptake *via* assimilating branches at night. Our results showed that *Ψ_sb_* > *Ψ_ab_* during sunny days in the rainfall pulse experiment (Figure 3A) and in outside-attached and outside-detached treatments in the high-humidity experiment (Figures 4C,D), which suggested that transpiration caused root absorption of soil water at night. In addition, stem water storage *via* a unique wood structure, i.e., internal secondary phloem through successive cambia, is considered an important strategy for plants surviving in a water-limited environment (Robert et al., 2014; Barraclough et al., 2018), including *H*. *ammodendron* (Li et al., 2015). Thus, the small amount of labeled water in the trunk stem might result from the dilution effect of stored water in the trunk stem. On the other hand, the absorbed atmospheric water was not transported from the assimilating branches to the taproot xylem and soils, likely because the foliar water uptake was a subsidiary strategy of desert plants such as *H. ammodendron* in adapting to extreme drought; and this secondary strategy might not be sufficient alone for sustaining plant growth (Yan et al., 2015). In this study, the basal diameter of the selected *H. ammodendron* individual in the high-humidity exposure experiment was 4.2 cm. Based on the previously established relationship between basal diameter of *H. ammodendron* and its root depth under similar growth conditions (Xu et al., 2016), the calculated root depth of the selected individual plant in the current study was enough to tap groundwater, in conformity with other reports that concluded that *H. ammodendron* was a groundwater-dependent desert plant (Liu et al., 2018; Wu et al., 2019). Thus, atmospheric water might merely be a subsidiary water resource in the sustainable survival of desert woody plants. Our results are consistent with a prior study that demonstrated that the atmospheric water of foliar uptake in coastal redwood *Sequoia sempervirens* (D. Don) only accounted for a small fraction of transpirational demand (Dawson, 1998; Burgess and Dawson, 2004). However, atmospheric water is of great significance to plants since the trees possess a relatively loose stomatal control of water loss, and increases in leaf surface wettability and water status could help suppress water loss from leaves (Burgess and Dawson, 2004). Atmospheric water may occupy a decisive position in the regulation of the water balance of plants despite low availability. Additionally, it was reported that atmospheric water would transport from leaves to rhizosphere soils and neighboring plants *via* the inverse and lateral hydraulic redistribution under long-term exposure to high humidity (Caldwell et al., 1998; Eller et al., 2013; Cassana et al., 2016). In that case, foliar water uptake might play important roles in spatial water redistribution and community maintenance. However, in this study, we did not detect labeled atmospheric water in rhizosphere soils of experimental and adjacent natural trees because the high-humidity exposure experiment only lasted one night. The long-term subsidiary benefits of foliar water uptake deserve more attention in future studies in arid desert regions. In addition, quantifying the proportion of atmospheric water in a plant is also an important aspect in analyzing its influences on ecosystem maintenance (Dawson, 1998; Kidron, 2000; Corbin et al., 2005).

Atmospheric water may play a role in regulating physiological performance through foliar water uptake (McHugh et al., 2015; Tomaszkiewicz et al., 2015). As an important indicator of plant water status (Elsayed et al., 2011), the improvement in water potential of the assimilating branches suggested that atmospheric water decreased water stress in the plant. Previous studies suggested that the hygroscopic atmospheric depositions on or around the stomata transform the leaf surface around the stomata from hydrophobic to hydrophilic, which could activate the stomata in assimilating branches of *H. ammodendron* during high-humidity periods (Burkhardt, 2010; Wang et al., 2016b). During this process, water transport through assimilating branches might result in some changes in water status and gas exchange. In this study, the predawn *Ψ*, *g*_s_, *E*, and WUEi in the assimilating branches differed between the inside and outside of the chamber (Figure 7). This indicated that foliar water uptake had an obvious relationship with physiological performance. In addition, traditional plant physiology theories demonstrate that plants could change their water transport-related traits to improve the capability of water absorption from soils to roots (Novák, 2012); thus, root water uptake also has an obvious relationship with water transport-related traits. In this study, foliar and root water uptake occurred simultaneously at night (Figures 5, 7), and our results showed that foliar water uptake, root water uptake and their interactions had significant influences on predawn *Ψ*, *g*_s_ and *E* (Figures 7A–C). The variability of WUEi was only determined by the foliar water uptake (Figure 7D) and may be due to the activation of stomata of assimilating branches inside the high-humidity chamber, which contributed to the water vaporization rate through transpiration (Ben-Asher et al., 2010; Wang et al., 2016b).

## Conclusions

The current study highlights that *H. ammodendron*, a tomentum-less and deep-rooted tree, could absorb atmospheric water, and a high level of atmospheric water was the prerequisite for such foliar water acquisition strategy. Our results also found that the absorbed atmospheric water was transported from the assimilating branches to the secondary branches and the trunk stems but not to the taproot xylem or the soil. This suggested that foliar water uptake of *H. ammodendron* had the opposite mechanism as the soil-plant-atmosphere system and traditional water physiology theories. In addition, the foliar and root water uptakes affected the water potential, stomatal conductance, transpiration rate, and instantaneous water use efficiency. These results indicated that foliar water uptake could result in variation in water status and gas exchange of *H. ammodendron*. Atmospheric water might be a kind of subsidiary water resource for the sustainable survival of deep-rooted desert trees with similar characteristics as *H. ammodendron* and warrants further research on this topic in various arid regions of the world.

## Author Contributions

X-WG and G-HL conceived and designed the experiments. X-WG and X-MH performed the experiments. X-WG and X-DY analyzed the data. X-WG, G-HL, BS, and X-DY wrote the manuscript.

### Conflict of Interest Statement

The authors declare that the research was conducted in the absence of any commercial or financial relationships that could be construed as a potential conflict of interest.

## References

[ref1] Barraclough A. D. Zweifel R. Cusens J. Leuzinger S. (2018). Daytime stem swelling and seasonal reversal in the peristaltic depletion of stored water along the stem of *Avicennia marina* (Forssk.) Vierh. Tree Physiol. 38, 965–978. 10.1093/treephys/tpy021, PMID: 29562284

[ref2] Ben-Asher J. Alpert P. Ben-Zvi A. (2010). Dew is a major factor affecting vegetation water use efficiency rather than a source of water in the eastern Mediterranean area. Water Resour. Res. 46:W10532. 10.1029/2008WR007484

[ref3] Breshears D. D. McDowell N. G. Goddard K. L. Dayem K. E. Martens S. N. Meter C. W.. (2008). Foliar absorption of intercepted rainfall improves woody plant water status most during drought. Ecology 89, 41–47. 10.1890/07-0437.1, PMID: 18376545

[ref4] Burgess S. S. O. Dawson T. E. (2004). The contribution of fog to the water relations of *Sequoia sempervirens* (d. don): foliar uptake and prevention of dehydration. Plant Cell Environ. 27, 1023–1034. 10.1111/j.1365-3040.2004.01207.x

[ref5] Burkhardt J. (2010). Hygroscopic particles on leaves: nutrients or desiccants? Ecol. Monogr. 80, 369–399. 10.1890/09-1988.1

[ref6] Caldwell M. M. Dawson T. E. Richards J. H. (1998). Hydraulic lift: consequences of water efflux from the roots of plants. Oecologia 113, 151–161. 10.1007/s004420050363, PMID: 28308192

[ref7] Cassana F. F. Eller C. B. Oliveira R. S. Dillenburg L. R. (2016). Effects of soil water availability on foliar water uptake of *Araucaria angustifolia*. Plant Soil 399, 147–157. 10.1007/s11104-015-2685-0, PMID: 18376545

[ref8] Corbin J. D. Thomsen M. A. Dawson T. E. D’antonio C. M. (2005). Summer water use by California coastal prairie grasses: fog, drought, and community composition. Oecologia 145, 511–521. 10.1007/s00442-005-0152-y 16001220

[ref9] Dai Y. Zheng X. J. Tang L. S. Li Y. (2015). Stable oxygen isotopes reveal distinct water use patterns of two *Haloxylon* species in the Gurbantonggut Desert. Plant Soil 389, 73–87. 10.1007/s11104-014-2342-z

[ref10] Dawson T. E. (1998). Fog in the California redwood forest: ecosystem inputs and use by plant. Oecologia 117, 476–485. 10.1007/s004420050683, PMID: 28307672

[ref11] Dawson T. E. Pate J. S. (1996). Seasonal water uptake and movement in root systems of Australian phraeatophytic plants of dimorphic root morphology: a stable isotope investigation. Oecologia 107, 13–20. 10.1007/BF00582230, PMID: 28307187

[ref12] Deng Y. B. Jiang Y. C. Liu J. (1998). The xeromorphic and saline morphic structure of leaves and assimilating branches in ten Chenopodiacea species in Xinjiang. Chin. J. Plant Ecol. 22, 164–170 (in Chinese with English abstract) http://www.plant-ecology.com/CN/Y1998/V22/I2/164

[ref13] Eller C. B. Lima A. L. Oliveira R. S. (2013). Foliar uptake of fog water and transport belowground alleviates drought effects in the cloud forest tree species, *Drimys brasiliensis* (Winteraceae). New Phytol. 199, 151–162. 10.1111/nph.12248, PMID: 23534879

[ref14] Eller C. B. Lima A. L. Oliveira R. S. (2016). Cloud forest trees with higher foliar water uptake capacity and anisohydric behavior are more vulnerable to drought and climate change. New Phytol. 211, 489–501. 10.1111/nph.13952, PMID: 27038126

[ref15] Elsayed S. Mistele B. Schmidhalter U. (2011). Can changes in leaf water potential be assessed spectrally? Funct. Plant Biol. 38, 523–533. 10.1071/FP11021 32480906

[ref16] Emery N. (2016). Foliar uptake of fog in coastal California shrub species. Oecologia 182, 731–742. 10.1007/s00442-016-3712-4, PMID: 27568025

[ref17] Goldsmith G. R. (2013). Changing directions: the atmosphere–plant–soil continuum. New Phytol. 199, 4–6. 10.1111/nph.12332, PMID: 23713550

[ref18] Golkarian A. Naghibi S. A. Kalantar B. Pradhan B. (2018). Groundwater potential mapping using C5.0, random forest, and multivariate adaptive regression spline models in GIS. Environ. Monit. Assess. 190:149. 10.1007/s10661-018-6507-8, PMID: 29455381

[ref19] Gotsch S. G. Asbjornsen H. Holwerda F. Goldsmith G. R. Weintraub A. E. Dawson T. E. (2014). Foggy days and dry nights determine crown-level water balance in a seasonal tropical Montane cloud forest. Plant Cell Environ. 37, 261–272. 10.1111/pce.12151, PMID: 23777598

[ref20] Grammatikopoulos G. Manetas Y. (1994). Direct absorption of water by hairy leaves of *Phlomis fruticosa* and its contribution to drought avoidance. Can. J. Bot. 72, 1805–1811. 10.1139/b94-222

[ref21] He X. M. Lv G. H. Qin L. Chang S. L. Yang M. Yang J. J.. (2015). Effects of simulated nitrogen deposition on soil respiration in a *Populous euphratica* community in the Ebinur Lake Area, a desert ecosystem of Northwestern China. PLoS One 10:e0137827. 10.1371/journal.pone.0137827, PMID: 26379186 PMC4575029

[ref22] Huang Z. Zhang X. Zheng G. Gutterman Y. (2003). Influence of light, temperature, salinity and storage on seed germination of *Haloxylon ammodendron*. J. Arid Environ. 55, 453–464. 10.1016/S0140-1963(02)00294-X

[ref23] Kidron G. J. (2000). Analysis of dew precipitation in three habitats within a small arid drainage basin, Negev Highlands, Israel. Atmos. Res. 55, 257–270. 10.1016/S0169-8095(00)00063-6

[ref200] Kidron G. J. (2010). The effect of substrate properties, size, position, sheltering and shading on dew: An experimental approach in the Negev Desert. Atmos. Res. 98, 378–386. 10.1016/j.atmosres.2010.07.015

[ref24] Li J. Wada H. Matsuzaki H. (2015). Radial growth rate through successive cambia in *Haloxylon ammodendron* (Chenopodiaceae) from the Gurbantunggut Desert, Northwestern China, determined by a series of radiocarbon dating. Geochem. J. 49, 39–51. 10.2343/geochemj.2.0328

[ref25] Liu Y. B. Li X. R. Li M. M. Liu D. Zhang W. L. (2016). Leaf (or assimilation branch) epidermal micromorphology of desert plant in arid and semiarid areas of China. Chin. J. Plant Ecol. 40, 1189–1207 (in Chinese with English abstract). 10.17521/cjpe.2016.0129

[ref26] Liu R. Wang Y. G. Li C. J. Ma J. Li Y. (2018). Partitioning water source and sinking process of a groundwater-dependent desert plant community. Plant Soil 430, 73–85. 10.1007/s11104-018-3714-6

[ref27] Ma H. Y. Yang X. D. Lü G. H. He X. M. Zhang X. N. Wang X. Y. (2017). Water resources of dominant desert species in Ebinur Wetland Nature Resure, Xinjiang, China. Acta Ecol. Sin. 37, 829–840 (in Chinese with English abstract). 10.5846/stxb201508311804

[ref28] Mastroberti A. A. de Araujo Mariath J. E. (2008). Development of mucilage cells of *Araucaria angustifolia* (Araucariaceae). Protoplasma 232, 233–245. 10.1007/s00709-007-0274-7, PMID: 18239849

[ref29] Mayr S. Schmid P. Laur J. Rosner S. Charra-Vaskou K. Dämon B.. (2014). Uptake of water via branches helps timberline conifers refill embolized xylem in late winter. Plant Physiol. 164, 1731–1740. 10.1104/pp.114.236646, PMID: 24521876 PMC3982737

[ref30] McHugh T. A. Morrissey E. M. Reed S. C. Hungate B. A. Schwartz E. (2015). Water from air: an overlooked source of moisture in arid and semiarid regions. Sci. Rep. 5:13767. 10.1038/srep13767, PMID: 26345615 PMC4561883

[ref31] Novák V. (2012). Evapotranspiration in the soil-plant-atmosphere system. Chapter 6. Movement of water in the soil root zone during transpiration. (Dordrecht: Springer).

[ref32] Oliveira R. S. Dawson T. E. Burgess S. S. O. (2005). Evidence for direct water absorption by the shoot of the desiccation-tolerant plant *Vellozia flavicans* in the savannas of central Brazil. J. Trop. Ecol. 21, 585–588. 10.1017/S0266467405002658

[ref33] Pan Z. Pitt W. G. Zhang Y. M. Wu N. Tao Y. Truscott T. T. (2016). The upside-down water collection system of *Syntrichia caninervis*. Nat. Plants. 2:16076. 10.1038/nplants.2016.76, PMID: 27302768

[ref34] Philip J. R. (1966). Plant water relations: some physical aspects. Annu. Rev. Plant Physiol. 17, 245–268. 10.1146/annurev.pp.17.060166.001333

[ref35] Pina A. L. C. B. Zandavalli R. B. Oliveira R. S. Martins F. R. Soares A. A. (2016). Dew absorption by the leaf trichomes of *Combretum leprosum* in the Brazilian semiarid region. Funct. Plant Biol. 43, 851–861. 10.1071/FP15337 32480509

[ref36] Robert E. M. R. Jambia A. H. Schmitz N. De Ryck D. J. R. De Mey J. Kairo J. G.. (2014). How to catch the patch? A dendrometer study of the radial increment through successive cambia in the mangrove *Avicennia marina*. Ann. Bot. 113, 741–752. 10.1093/aob/mcu001, PMID: 24510216 PMC3936594

[ref37] Schönherr J. (2006). Characterization of aqueous pores in plant cuticles and permeation of ionic solutes. J. Exp. Bot. 57, 2471–2491. 10.1093/jxb/erj217, PMID: 16825315

[ref38] Schwerbrock R. Leuschner C. (2017). Foliar water uptake, a widespread phenomenon in temperate woodland ferns? Plant Ecol. 218, 555–563. 10.1007/s11258-017-0711-4

[ref39] Steppe K. Vandegehuchte M. W. Van de Wal B. A. E. Hoste P. Guyot A.. (2018). Direct uptake of canopy rainwater causes turgor-driven growth spurts in the mangrove *Avicennia marina*. Tree Physiol. 38, 979–991. 10.1093/treephys/tpy024, PMID: 29562244

[ref40] Tobe K. Li X. Omasa K. (2000). Effects of sodium chloride on seed germination and growth of two Chinese desert shrubs, *Haloxylon ammodendron* and *H. persicum* (Chenopodiaceae). Aust. J. Bot. 48, 455–460. 10.1071/BT99013

[ref41] Tomaszkiewicz M. Abou Najm M. Beysens D. Alameddine I. Elfadel M. (2015). Dew as a sustainable non-conventional water resource: a critical review. Environ. Rev. 23, 425–442. 10.1139/er-2015-0035

[ref42] Vitarelli N. C. Riina R. Cassino M. F. Merira R. M. S. A. (2016). Trichome-like emergences in *Croton* of Brazilian highland rock outcrops: evidences for atmospheric water uptake. Perspect. Plant Ecol. Evol. Syst. 22, 23–35. 10.1016/j.ppees.2016.07.002

[ref43] Wang X. H. Xiao H. L. Cheng Y. B. Ren J. (2016a). Leaf epidermal water-absorbing scales and their absorption of unsaturated atmospheric water in *Reaumuria soongorica*, a desert plant from the northwest arid region of China. J. Arid Environ. 128, 17–29. 10.1016/j.jaridenv.2016.01.005

[ref44] Wang X. H. Xiao H. L. Ren J. Cheng Y. B. Qiu Y. (2016b). An ultrasonic humidification fluorescent tracing method for detecting unsaturated atmospheric water absorption by the aerial parts of desert plants. J. Arid Land 8, 272–283. 10.1007/s40333-015-0018-z

[ref45] Westhoff M. Zimmermann D. Gessner P. Wegner L. H. Bentrup F. W. Zimmermann U. (2009). Distribution and function of epistomatal mucilage plugs. Protoplasma 235, 101–105. 10.1007/s00709-008-0029-0, PMID: 19145400

[ref46] Wu X. Zheng X. J. Li Y. Xu G. Q. (2019). Varying responses of two *Haloxylon* species to extreme drought and groundwater depth. Environ. Exp. Bot. 158, 63–72. 10.1016/j.envexpbot.2018.11.014

[ref47] Xu H. Li Y. Xu G. Q. Zou T. (2007). Ecophysiological response and morphological adjustment of two Central Asian desert shrubs towards variation in summer precipitation. Plant Cell Environ. 30, 399–409. 10.1111/j.1365-3040.2006.001626.x, PMID: 17324227

[ref48] Xu G. Q. Mcdowell N. G. Li Y. (2016). A possible link between life and death of a xeric tree in desert. J. Plant Physiol. 194, 35–44. 10.1016/j.jplph.2016.02.014, PMID: 26968083

[ref49] Yan X. Zhou M. Dong X. Zou S. B. Xiao H. L. Ma X. F. (2015). Molecular mechanisms of foliar water uptake in a desert tree. AoB Plants 7:plv129. 10.1093/aobpla/plv129 26567212 PMC4685171

[ref50] Yang X. D. Lv G. H. Ali A. Ran Q. Y. Gong X. W. Wang F. (2017). Experimental variations in functional and demographic traits of *Lappula semiglabra* among dew amount treatments in an arid region. Ecohydrology 10:e1858. 10.1002/eco.1858

[ref51] Yang X. D. Zhang X. N. Lv G. H. Ali A. (2014b). Linking *Populus euphratica* hydraulic redistribution to diversity assembly in the Arid Desert Zone of Xinjiang, China. PLoS One 9:e109071. 10.1371/journal.pone.0109071 25275494 PMC4183514

[ref52] Yang Q. Y. Zhao W. Z. Liu B. Liu H. (2014a). Physiological responses of *Haloxylon ammodendron* to rainfall pulses in temperate desert regions, Northwestern China. Trees Struct. Funct. 28, 709–722. 10.1007/s00468-014-0983-4

[ref53] Zheng X. Q. Zheng X. J. Li Y. (2012). Distribution and change of different precipitation pulse size in the southern marginal zone of the Junggar Basin, China. Arid Zone. Res. 29, 495–502 (in Chinese with English abstract). 10.1007/s11783-011-0280-z

[ref54] Zhuang Y. L. Ratcliffe S. (2012). Relationship between dew presence and *Bassia dasyphylla* plant growth. J. Arid Land 4, 11–18. 10.3724/SP.J.1227.2012.00011

[ref55] Zhuang Y. L. Zhao W. Z. (2017). Dew formation and its variation in *Haloxylon ammodendron* plantations at the edge of a desert oasis, northwestern China. Agr. For. Meteorol. 247, 541–550. 10.1016/j.agrformet.2017.08.032

[ref56] Zimmermann U. Schneider H. Wegner L. H. Haase A. (2004). Water ascent in tall trees: does evolution of land plants rely on a highly metastable state? New Phytol. 162, 575–615. 10.1111/j.1469-8137.2004.01083.x 33873767

[ref57] Zimmermann D. Westthoff M. Zimmermann G. Geßner P. Gessner A. Wegner L. H.. (2007). Foliar water supply of tall trees: evidence for mucilage-facilitated moisture uptake from the atmosphere and the impact on pressure bomb measurements. Protoplasma 232, 11–34. 10.1007/s00709-007-0279-2, PMID: 18176835

